# Co-effects of m6A and chromatin accessibility dynamics in the regulation of cardiomyocyte differentiation

**DOI:** 10.1186/s13072-023-00506-6

**Published:** 2023-08-11

**Authors:** Xue-Hong Liu, Zhun Liu, Ze-Hui Ren, Hong-Xuan Chen, Ying Zhang, Zhang Zhang, Nan Cao, Guan-Zheng Luo

**Affiliations:** 1 MOE Key Laboratory of Gene Function and Regulation, Guangdong Province Key Laboratory of Pharmaceutical Functional Genes, State Key Laboratory of Biocontrol, School of Life Sciences, Sun Yat-sen University, Guangzhou, 510275 China; 2 Zhongshan School of Medicine, Sun Yat-sen University, No.74 Zhongshan Rd.2, Guangzhou, 510080 China

**Keywords:** m6A, Cardiomyocyte differentiation, Chromatin accessibility

## Abstract

**Background:**

Cardiomyocyte growth and differentiation rely on precise gene expression regulation, with epigenetic modifications emerging as key players in this intricate process. Among these modifications, N6-methyladenosine (m6A) stands out as one of the most prevalent modifications on mRNA, exerting influence over mRNA metabolism and gene expression. However, the specific function of m6A in cardiomyocyte differentiation remains poorly understood.

**Results:**

We investigated the relationship between m6A modification and cardiomyocyte differentiation by conducting a comprehensive profiling of m6A dynamics during the transition from pluripotent stem cells to cardiomyocytes. Our findings reveal that while the overall m6A modification level remains relatively stable, the m6A levels of individual genes undergo significant changes throughout cardiomyocyte differentiation. We discovered the correlation between alterations in chromatin accessibility and the binding capabilities of m6A writers, erasers, and readers. The changes in chromatin accessibility influence the recruitment and activity of m6A regulatory proteins, thereby impacting the levels of m6A modification on specific mRNA transcripts.

**Conclusion:**

Our data demonstrate that the coordinated dynamics of m6A modification and chromatin accessibility are prominent during the cardiomyocyte differentiation.

**Supplementary Information:**

The online version contains supplementary material available at 10.1186/s13072-023-00506-6.

## Introduction

Heart diseases have long been one of the leading causes of death worldwide [1, 2]. However, due to the scarcity of effective treatments, the primary approaches for addressing heart diseases are organ transplantation and cell therapy [3–5]. At present, the ability to generate cardiomyocytes (CMs) from human pluripotent stem cells (hPSCs) has not yet met the demands of cell therapy [6–8]. As a result, understanding the mechanisms underlying cardiomyocyte differentiation is critical for enhancing the efficiency of this process and for the treatment of heart diseases [9–11].

Emerging research has highlighted the importance of epigenetic modifications in regulating cardiomyocyte differentiation, including DNA methylation and histone modifications [12–14]. Recent studies show that chemical modifications on RNA play another layer of epigenetic regulation. Among over 150 known RNA modifications, m6A is the most prevalent found in mammalian mRNAs [15]. It is primarily added to mRNA by a methyltransferase complex, composed of METTL3, METTL14, and auxiliary proteins, and removed by demethylases FTO and ALKBH5 [16–19]. The m6A modification can be recognized by various reader proteins, including YTHDF1-3 and YTHDC1-2. These reader proteins bind to an m6A site to carry out their biological functions [20–23]. The interplay of writers, erasers, and readers on m6A makes the modification highly dynamic, playing a crucial role in mRNA metabolism involves alternative splicing, nuclear export, translation, and degradation.

Recent studies have demonstrated that the m6A modification significantly impacts various biological processes, including cell differential, disease development, neurodevelopment, and immunity [24]. Perturbations in m6A levels can detrimentally affect cellular proliferation capacity, leading to unfavorable outcomes for organisms [25]. Different differentiation systems demonstrate distinct patterns of m6A level changes [26, 27]. For instance, investigations have revealed relatively minor dynamic changes in m6A levels during hematopoietic stem cell differentiation, whereas substantial variations in m6A levels have been observed during neural cell differentiation [26, 27]. This also holds true for cardiomyocyte differentiation, where ALKBH5 can effectively influence the regenerative capacity of the heart [28]. However, due to limitations in m6A detection methods, it has been challenging to achieve high-throughput, accurate, and quantitative m6A detection in the past [29, 30]. These limitations have hindered our ability to comprehensively investigate how m6A modification changes during the process of differentiation, impeding our understanding of the functional relevance and underlying mechanisms of m6A in various differentiation processes.

In this study, we employed a quantitative m6A detection method by performing multiplexed m6A-immunoprecipitation on barcoded and pooled samples [31]. This approach allowed us to obtain a precise m6A profiling map at various stages of cardiomyocyte differentiation. We observed dynamic changes in m6A levels across genes involved in cardiomyocyte differentiation, highlighting the potential role of m6A in orchestrating this process. Notably, we identified specific target genes of m6A writers and erasers that contribute to the regulation of RNA translation during cardiomyocyte differentiation, suggesting their involvement in modulating the progression of differentiation. We explored the interplay between m6A modification and chromatin accessibility and discovered that the binding capabilities of m6A writers and erasers may depend on accessible chromatin regions. Our findings indicate that chromatin accessibility influences the distribution of m6A modifications, with different reader proteins exhibiting distinct responses to m6A modifications depending on the chromatin accessibility context. This suggests a synergistic relationship between m6A modification and chromatin accessibility in regulating early cardiomyocyte differentiation. Furthermore, we observed that m6A modification could independently regulate the transition of cardiac progenitor cells to cardiomyocytes, highlighting the unique role of m6A in specific stage of differentiation. These findings contribute to the broader understanding of epigenetic regulation in cardiac development and provide potential avenues for therapeutic interventions targeting cardiomyocyte differentiation in the future.

## Results

### Overall m6A levels are stable during cardiomyocyte differentiation

**Fig. 1:**
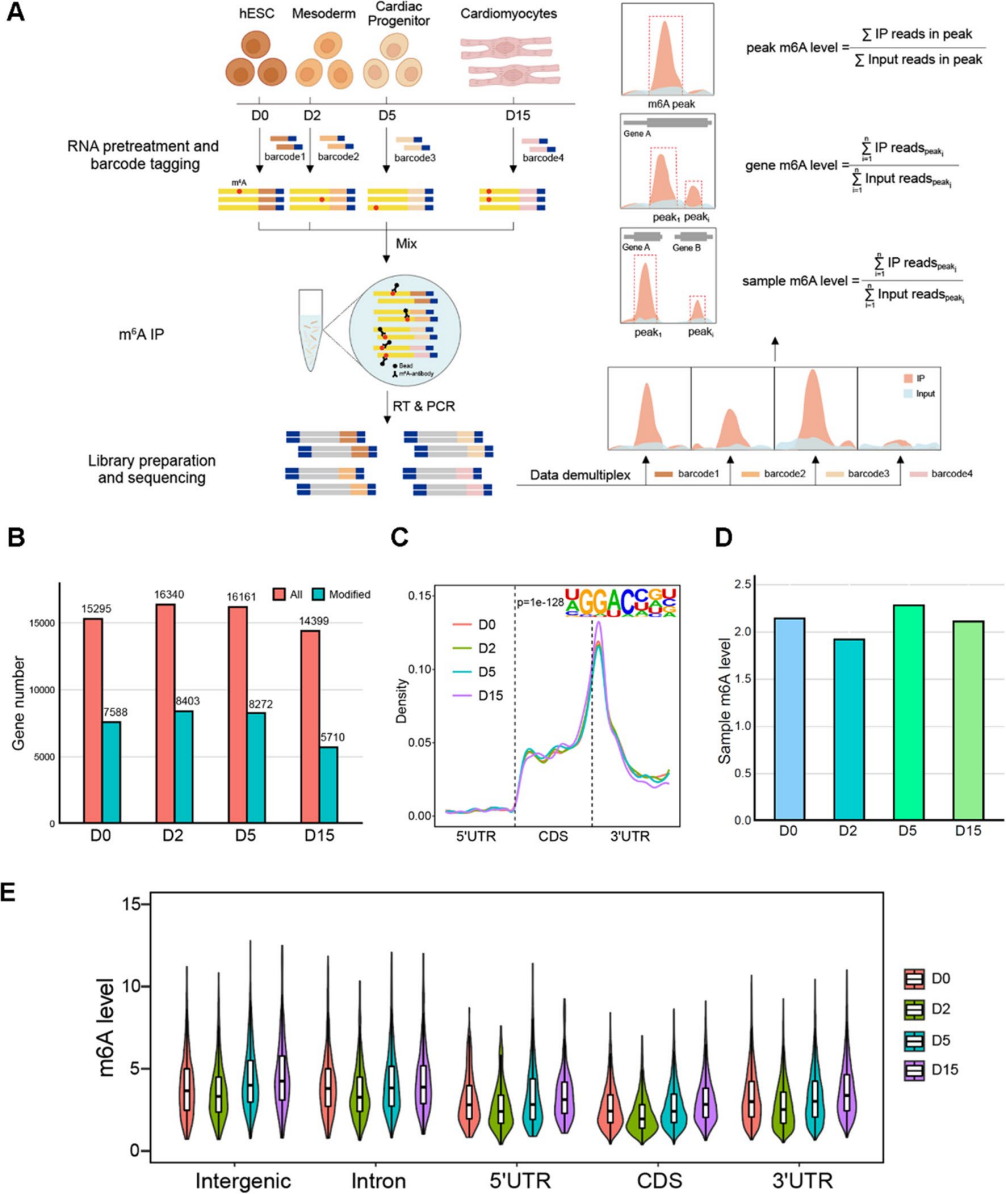
General features of m6A modification during cardiomyocyte differentiation. **A** Schematic overview of the study design. **B** Number of expressed genes and m6A-modifed genes detected at four stages during cardiomyocyte differentiation. **C** Distribution of m6A peaks on transcripts and enriched motifs during cardiomyocyte differentiation. **D** m6A levels (IP/input ratio) of four stages during cardiomyocyte differentiation. **E** m6A levels in different genetic regions during cardiomyocyte differentiation. D0, Day0; D2, Day2; D5, Day5, D10, Day10

To obtain an unbiased m6A profile map during human cardiomyocyte differentiation, we first induced human pluripotent stem cells to differentiate into cardiomyocytes. We collected sequential samples at different stages: human Embryonic Stem Cells (hESCs) at day 0 (D0), Mesoderm at day 2 (D2), Cardiac Progenitor Cells at day 5 (D5), and fully differentiated Cardiomyocytes at day 15 (D15). The collected samples were then divided into two portions, with one portion used for RNA-seq analysis and the other for MeRIP-seq analysis. For precise and quantitative assay, we employed MeRIP-seq with a multiplexed m6A-immunoprecipitation strategy on barcoded and pooled samples [31]. Each sample was uniquely barcoded and then combined for immunoprecipitation (IP) using the m6A antibody. This approach effectively eliminated deviations in relative m6A quantification arising from variations in IP efficiency, resulting in more accurate and unbiased m6A profiles [31]. Following the barcoding, each sample was easily distinguishable based on its unique barcode. We then analyzed the coverage of immunoprecipitated (IP) reads and input reads within the m6A peaks of each sample. By calculating the ratio of IP reads to input reads, we determined the relative m6A levels of the peaks. The m6A level of each gene was defined as the overall methylation level across all m6A peaks associated with that gene. Moreover, the m6A levels of a specific sample represented the cumulative methylation level across all m6A peaks within that particular sample (Fig. 1A).

To validate the successful differentiation of cardiomyocytes, we analyzed the RNA-seq data and observed that the expression of stage-specific marker genes was highly elevated, consistent with the expected patterns [32–34] (Additional file 1: Fig. S1A, B). We next examined the overall m6A levels throughout the differentiation process. Remarkably, the proportion of m6A-modified genes in the cells remained relatively stable, with approximately one-third of the genes exhibiting m6A modification (Fig. 1B). Moreover, the distribution of m6A on transcripts mirrored previous findings, predominantly occurring within the coding sequence (CDS) and 3′ untranslated region (3′ UTR) regions, with a notable enrichment near stop codons. These m6A-enriched regions exhibited a distinct preference for the DRACH motif, and this distribution pattern remained consistent as differentiation progressed (Fig. 1C).

The multiplexed m6A-immunoprecipitation strategy also allowed us to estimate the overall m6A methylation level of each specific sample [31]. We further compared the overall m6A levels across the four differentiation stages. In contrast to other epigenetic modifications such as 5 mC, which undergo substantial changes during cellular reprogramming, we observed only a slight decrease in m6A levels from D0 to D2 and an insignificant increase from D2 to D5. Notably, the m6A level of D15 returned to a level comparable to that of D0 (Fig. 1D). This subtle variation pattern was also observed for m6A at different gene locations (Fig. 1E). These findings suggest that despite the thorough reprogramming of cellular identity, the overall m6A modification levels remained relatively stable during cardiomyocyte differentiation.

### Genes involved in cardiomyocyte differentiation undergo changes at the m6A level

**Fig. 2:**
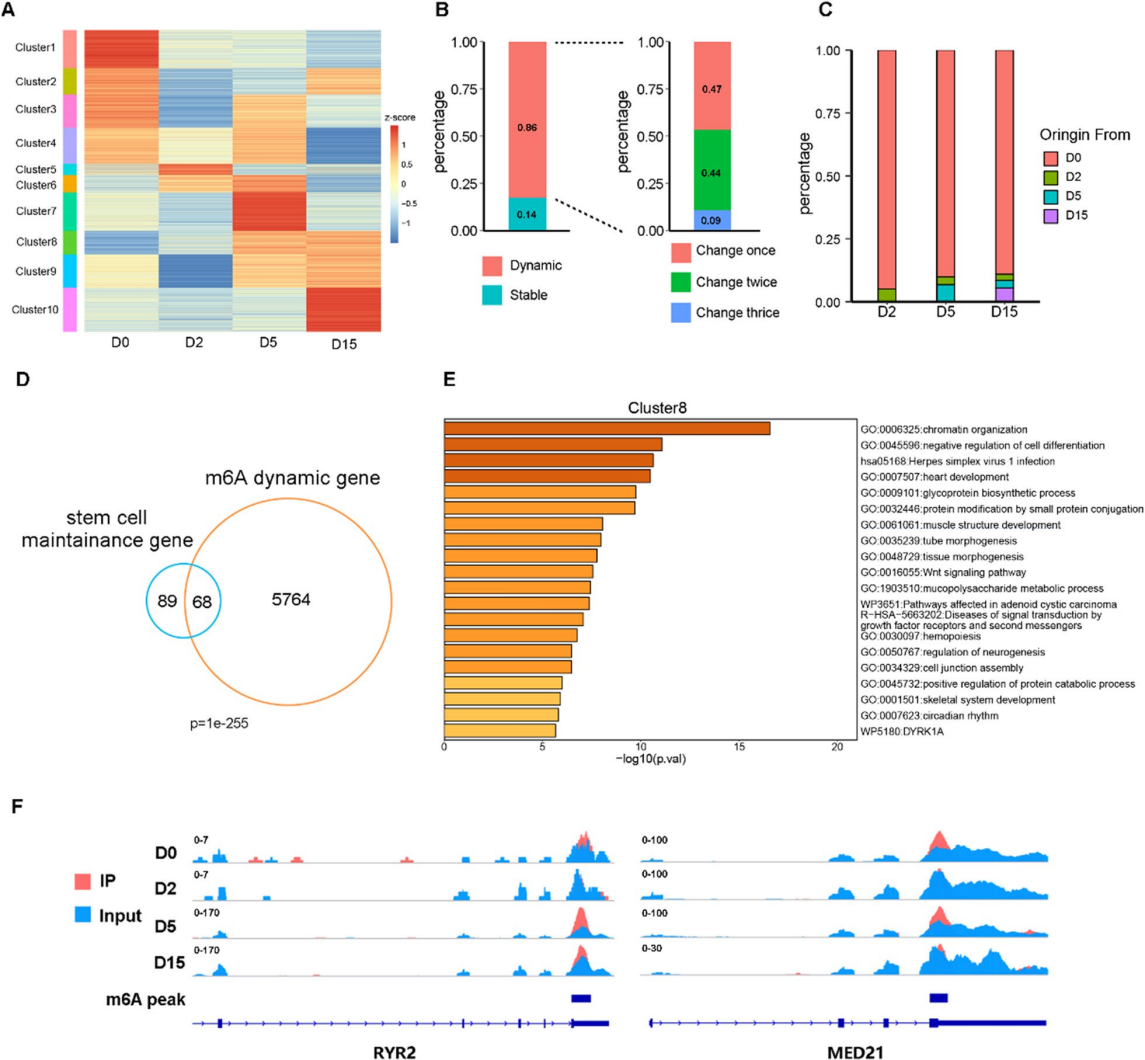
Dynamics of m6A during cardiomyocyte differentiation. **A** Heatmap of m6A level for each peak at four differentiation stages, clustered using k-means. **B** The percentages of m6A with changes (Fold Change > 1.2, red bar) and without changes (blue bar). Right: The peaks that show changes in m6A modification levels on the left are further subdivided into peaks that change once, twice and thrice. **C** Origin of m6A peaks at various stages during cardiomyocyte differentiation. **D** The genes related to stem cell maintenance obtained from the GO database were compared with the genes that showed m6A modifications. The p-value was calculated using a one-tailed hypergeometric test. (E) GO enrichment analysis results for the gene sets contained in cluster 8 in A. **F** Genome browser view of the m6A signal at four differentiation stages on RYR2 and MED21

Although no obvious overall dynamic changes in m6A modification were observed, we considered whether m6A modifications in certain genes could affect cardiomyocyte differentiation [35, 36]. To address this question, we further analyzed changes in the m6A peak modification levels at different differentiation stages. In contrast to the overall level, we found that only about 14% of the peaks maintained a constant m6A level when examining changes in m6A modification levels at the peak level. The remaining 86% of the peaks exhibited changing m6A levels throughout the differentiation process, with approximately 9% of peaks showing changes in m6A levels in all differentiation stages (Fig. 2A, B). We reasoned that the changes in m6A levels could occur through the gaining or losing of m6A modification during differentiation, which may reflect underlying regulatory mechanics. To investigate this, we calculated the ratio of IP reads to input reads in regions with m6A peaks. We defined regions with a ratio greater than 1.5 as having m6A modifications and those with a ratio less than 1.5 as not having m6A modification. Using this definition, we counted the number of peaks where m6A was gained or lost at each differentiation stage. Our analysis revealed that approximately 90% of the peaks had already been modified at the stem cell stage, while very few of new m6A peaks emerged during the subsequent differentiation process (Fig. 2C). These findings suggest that the m6A loci relevant to cardiomyocyte differentiation are pre-determined as early as the stem cell stage, rather than being established during the subsequent differentiation process in response to differentiation cues.

Next, we examined whether these altered m6A peaks are associated with cardiomyocyte differentiation. We identified genes that showed larger changes in m6A levels (fold change > 1.2) at least once during the process of cardiomyocyte differentiation and referred to them as dynamic m6A genes. We discovered that the dynamic m6A genes tended to have a high overlap with genes related to the cardiomyocyte differentiation process (Fig. 2D). Based on the clustered peaks according to their m6A levels (Fig. 2A), we performed GO enrichment analysis on gene sets in different clusters. The results indicated that genes with m6A level changes were indeed associated with cardiomyocyte differentiation (Fig. 2E, Additional file 1: Fig. S2). Taking the cardiomyocyte-related gene RYR2 and stem cell-related gene MED21 as an example, it was clear that the m6A peak modification level significantly decreased from D0 to D2, increased to a higher level at D5, and finally returned to a state similar to D0 at D15 (Fig. 2F). These dynamic changes of m6A level may have influences on the cardiomyocyte differentiation.

### The expression changes of m6A writer, reader and eraser contribute to the cardiomyocyte differentiation

**Fig. 3:**
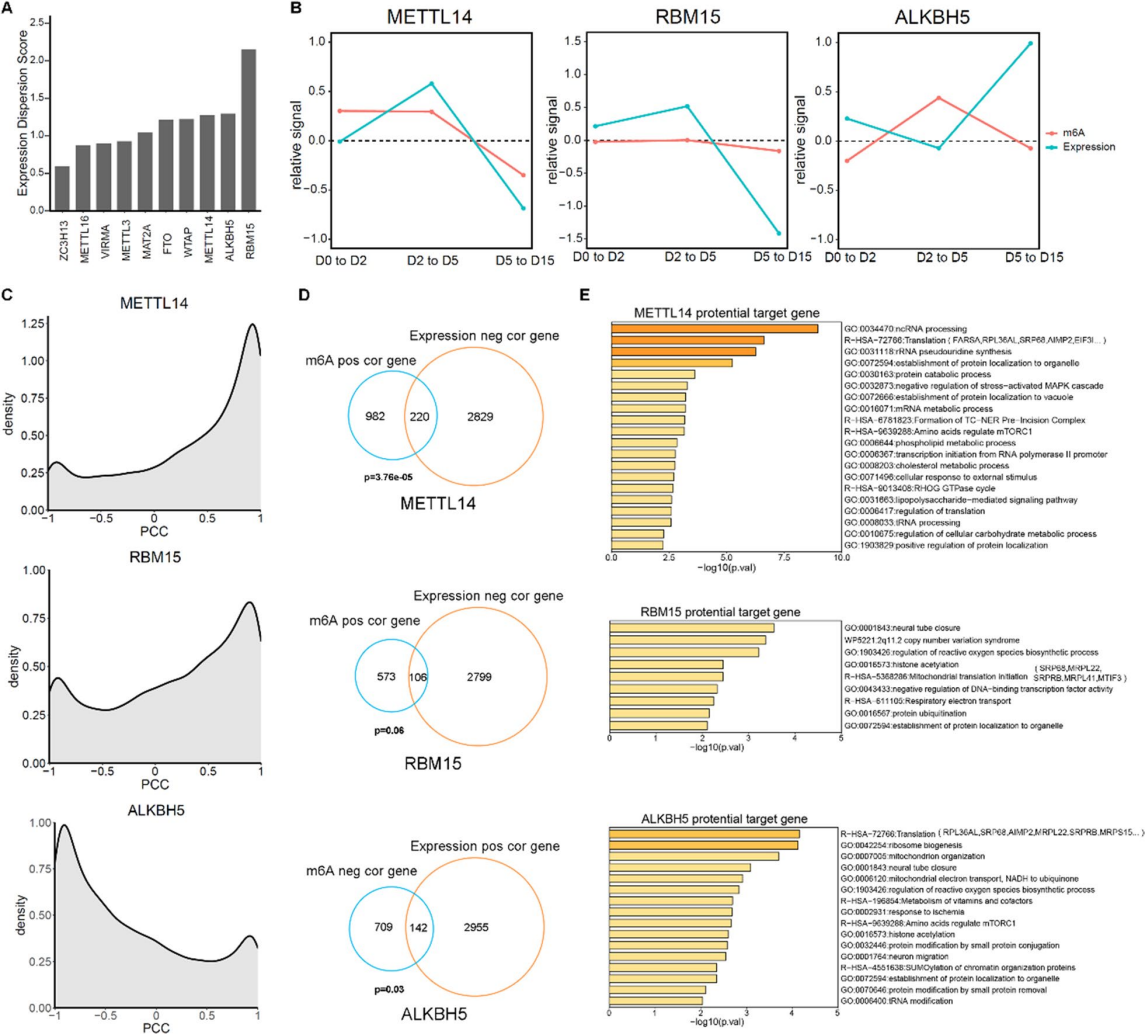
Dynamics of METTL14, RBM15, ALKBH5 during cardiomyocyte differentiation. **A** Dispersion score of expression levels of m6A writers and erasers during cardiomyocyte differentiation. **B** Changes in expression levels (blue line) and m6A modification levels (red line) of METTL14, RBM15, ALKBH5 during differentiation. The relative signal represents the change in the latter stage compared to the previous stage. **C** Distribution of the correlation between the m6A level of the transcriptome and the expression of METTL14, RBM15, and ALKBH5. PCC: Pearson correlation coefficient. **D** Identification of potential target genes of METTL14, RBM15, ALKBH5. m6A pos/neg cor gene: genes whose m6A levels are positively/negatively correlated with the expression of m6A-related proteins. Expression pos/neg cor gene: genes whose expression levels are positively/negatively correlated with the expression of m6A-related proteins. The p-value was calculated using one-tailed hypergeometric test. (E) GO enrichment analysis of potential target genes of METTL14, RBM15 and ALKBH5

To explore the underlying mechanisms behind the dynamics of m6A during cardiomyocyte differentiation, we examined the expression changes of known m6A writers and erasers. Our results revealed that the m6A writers METTL14 and RBM15, as well as the eraser ALKBH5, showed a higher degree of expression change (Fig. 3A), suggesting that these three genes may play a role in regulating the changes in m6A levels during cardiomyocyte differentiation. Considering that their own expression may be affected by the m6A level on the corresponding mRNA (Additional file 1: Fig. S3A, B), we compared the expression changes of these genes with the m6A level changes. The results revealed that, compared to the expression level, the dynamic changes of m6A levels on these genes were relatively weak (Fig. 3B), implying that the changes in the expression levels of these genes were influenced by other factors rather than regulated by their own m6A sites. Additionally, we sought to confirm whether the expression level could represent the function of these genes. To this end, we performed a correlation analysis on the expression levels of these genes and the m6A level of the entire transcriptome. As expected, for the writers METTL14 and RBM15, the expression level was primarily positively correlated with the m6A level of the entire transcriptome, while the expression level of ALKBH5, an eraser, was mainly negatively correlated with the m6A level of the transcriptome (Fig. 3C). This indicates that, to a certain extent, we can use the expression level of these genes to represent their functional strength.

Next, we aimed to identify which genes were most likely to be regulated as the target genes of METTL14, RBM15, and ALKBH5. To achieve this, we analyzed the expression levels and the m6A levels of the entire transcriptome, correlating them with the expression levels of these three genes, respectively. m6A modifications has been demonstrated to negatively regulate gene expression through an accelerated RNA decay mechanism. Given that the m6A writer proteins, such as METTL14 and RBM15, are responsible for adding the m6A modification, which can lead to a decrease in gene expression, we focused on identifying genes that exhibited the most negative correlation with the expression levels of METTL14 or RBM15. Additionally, we identified genes with the highest positive correlation with the m6A levels mediated by these two writers. The intersection of these two groups was suggested to be the genes that serve as potential target genes for the writers. Conversely, we identified a set of genes that overlapped between those exhibiting the highest positive correlation with gene expression and those showing the most negative correlation with m6A levels mediated by the m6A eraser ALKBH5. These genes were considered as potential targets of ALKBH5-mediated regulation. Using this approach, we identified 220, 106, and 142 potential target genes corresponding to METTL14, RBM15, and ALKBH5, respectively (Fig. 3D). Next, we performed GO enrichment analysis on these potential target genes of the identified m6A writers and erasers. Intriguingly, we observed that the common GO terms shared by three genes were predominantly associated with RNA metabolism and translation (Fig. 3E). This finding suggests that the writers and erasers of m6A modifications co-regulate the transition of cell fate during cardiomyocyte differentiation by modulating mRNA and protein expression levels through the regulation of m6A modifications.

Given that the regulatory function of m6A is largely dependent on m6A readers, we further investigated whether the expression levels of these cardiogenesis-related genes targeted by writers or erasers were associated with the expression of known readers. Interestingly, we observed that the expression levels of different genes exhibited varying correlations with the expression levels of various readers (Additional file 1: Fig. S4). For example, the expression level of the target gene FOXO1 showed a stronger correlation with the expression level of reader YTHDF2. This finding suggests that YTHDF2 is more likely to recognize and interact with FOXO1, subsequently exerting downstream functions in cardiomyocyte differentiation. These findings highlight the complex interplay between m6A writers, erasers, and readers in orchestrating gene expression dynamics during cardiomyocyte differentiation.

### Chromatin accessibility affects the function of m6A regulators

**Fig. 4:**
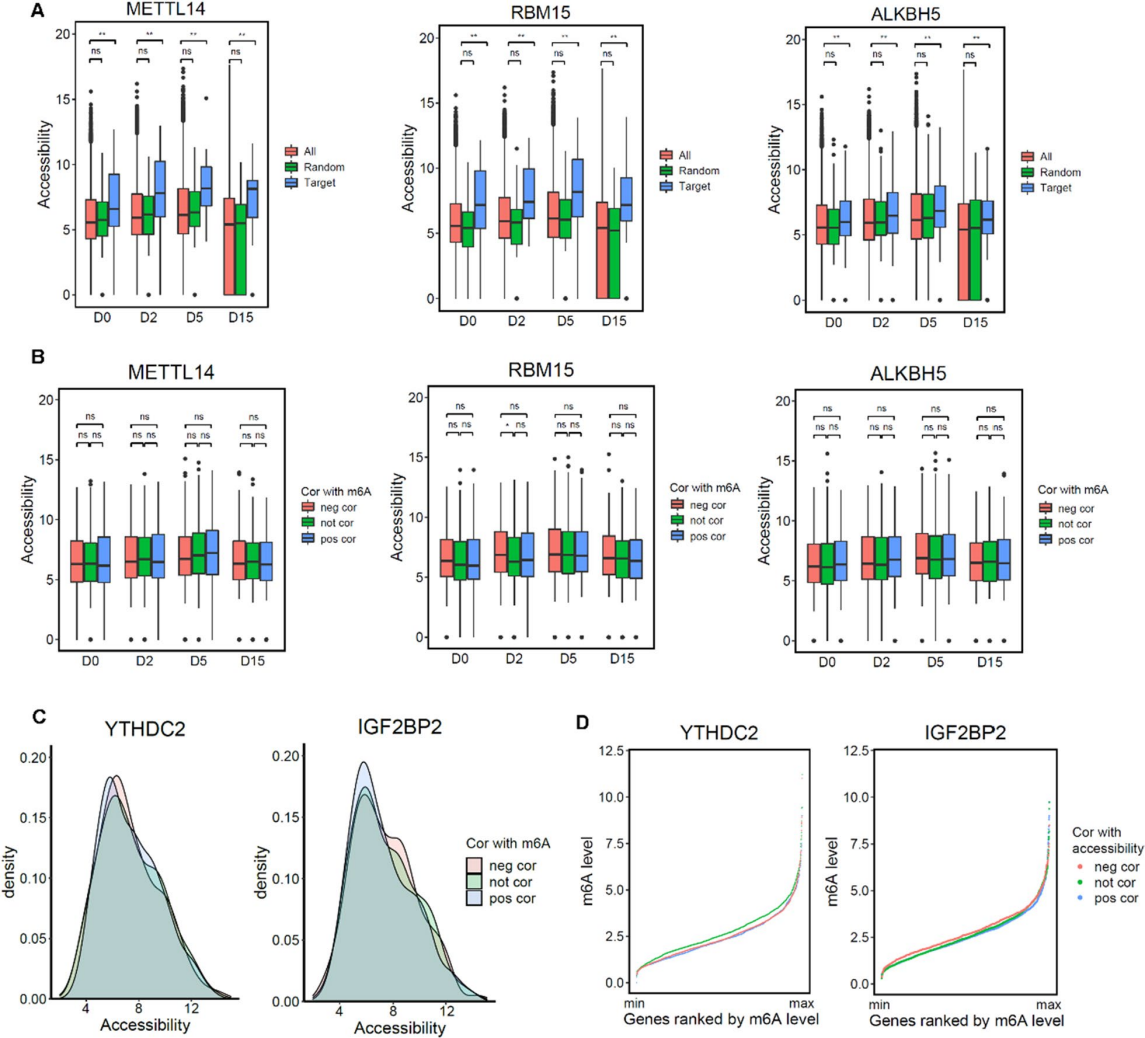
Relationship between chromatin accessibility and m6A-related proteins. **A** The accessibility of potential target genes of m6A-related proteins compared with the accessibility of all genes. Blue represents potential target genes, green represents random genes with same number as potential target genes and red represents all genes we detected. * represents p-value < 0.05 and ** represents p-value < 0.01. **B** Genes exhibiting various correlations between m6A level and the expression level of METTL14, RBM15 and ALKBH5 show similar accessibility. * represents p-value < 0.05. **C** Genes exhibiting various correlations between m6A level and the expression level of YTHDC2 and IGF2BP2 show similar accessibility. **D** Cumulative curve shows the m6A modification levels of genes displaying different correlations between m6A modification levels and expression levels of YTHDC2 and IGF2BP2. neg/not/pos cor: genes whose m6A level or accessibility are negatively/not/positively correlated with the expression of m6A-related proteins

Intrigued by the previous findings on changes in chromatin accessibility during cardiomyocyte differentiation [37, 38], we aimed to explore the potential influence of m6A regulator proteins in this process through their interaction with chromatin accessibility. To investigate this, we harnessed the power of ATAC-seq data [38] that included samples from ESC, Mesoderm, Cardiac Progenitor, and Cardiomyocytes. Our analysis revealed a compelling observation that the potential target genes of m6A writer and eraser proteins exhibited significantly higher levels of chromatin accessibility compared to non-target genes (Fig. 4A, p-value < 0.05). This intriguing finding suggests that a more permissive chromatin structure, characterized by enhanced accessibility, may facilitate the recruitment and binding of m6A writer and eraser proteins to the regulatory regions of m6A-modified genes. We further examined the relationship between the levels of chromatin accessibility and m6A in these target genes. We found that although enhanced chromatin accessibility may facilitate the establishment of m6A modification, the level of m6A was determined independently of the level of chromatin accessibility (Fig. 4B). Furthermore, we extended our analysis to include known m6A readers and observed that the effects of readers did not significantly influence the accessibility level neither (Fig. 4C, Additional file 1: Fig. S5A). Reversely, we asked whether changes in chromatin accessibility affect the function of readers. We classified genes into three categories based on the correlation between reader expression and chromatin accessibility: positive correlation, no correlation, and negative correlation. We observed distinct patterns of m6A levels among these gene categories, suggesting that changes in chromatin accessibility may indeed impact the function of m6A reader proteins. For instance, genes with accessibility levels not correlated with YTHDC2 expression exhibited a higher degree of m6A, potentially implying that YTHDC2 binding on low-level modified m6A genes is more sensitive to chromatin state (Fig. 4D). On the other hand, genes with accessibility levels positively correlated with IGF2BP2 expression displayed lower m6A levels, which could be due to accessible chromatin making it easier for IGF2BP2 to bind to its targets (Fig. 4D). In addition to these two readers, some other readers also show certain chromatin accessibility associations (Additional file 1: Fig. S5B). This suggests that for proteins involved in m6A-related functions, chromatin accessibility may indeed impact their functionality.

### The co-effect of chromatin accessibility and m6A is stage-specific

**Fig. 5:**
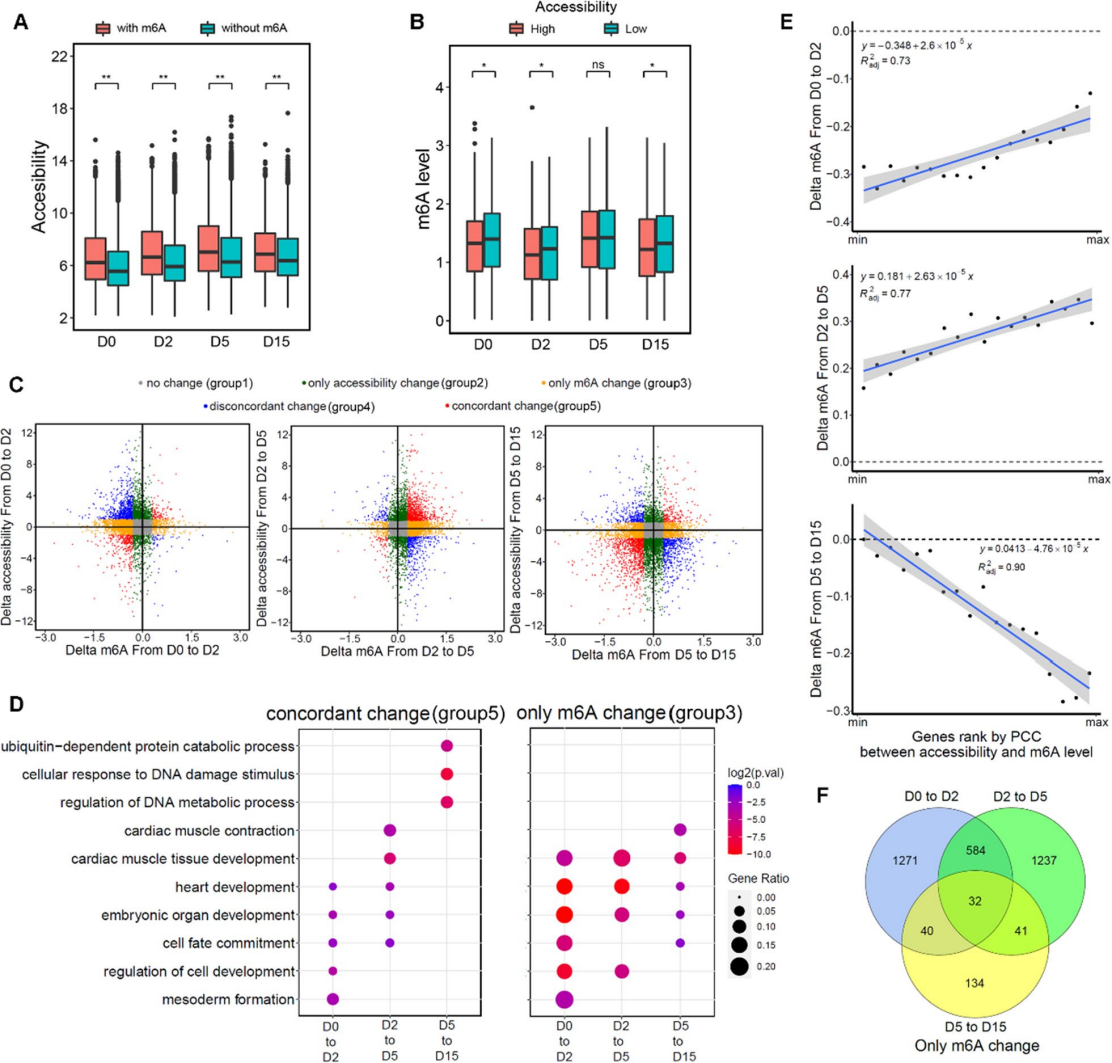
The relationship between the chromatin accessibility and m6A level. **A** Chromatin accessibility of genes with(red) and without(blue) m6A modification. * represents p-value < 0.05, Wilcoxon test. **B** Comparison of m6A levels in genes with varying chromatin accessibility. Genes were classified into three categories based on their chromatin accessibility: the top 10% of genes with high accessibility, the bottom 10% of genes with low accessibility, and the remaining genes with medium accessibility. * represents p-value < 0.05, Wilcoxon test. **C** Changes in m6A level and chromatin accessibility during differentiation, with colors indicating 5 gene categories (group 1 to group 5) based on their distinct patterns of changes. **D** GO enrichment analysis results for gene categories from Fig. 5C. **E** Relationship between the correlation of m6A levels and accessibility, and m6A level changes at various differentiation stages. Genes were ranked according to the PCC of their m6A levels with chromatin accessibility, and every 5% of genes were grouped without top 5% and bottom 5%. PCC: Pearson correlation coefficient. **F** Overlap of genes with only m6A change across different differentiation stages

In light of previous findings, we speculated that chromatin accessibility changes during cardiomyocyte differentiation could impact the functions of m6A-related proteins. Consequently, we hypothesized that the chromatin accessibility could positively or negatively correlated with m6A levels of corresponding genes, thereby co-regulate cardiomyocyte differentiation. To test this, we first compared chromatin accessibility between genes with and without m6A modification. Our findings revealed that genes with m6A modification exhibited higher chromatin accessibility than those without modification (Fig. 5A), consistent with our earlier observation that writer and eraser target genes have higher chromatin accessibility. However, for genes with m6A modification, higher chromatin accessibility correlated with lower m6A levels (Fig. 5B; Wilcoxon test, P-value < 0.05). These results suggest that chromatin accessibility likely influences m6A levels. Given that chromatin opening is often accompanied by transcription factor binding, and some transcription factors play crucial roles in cardiomyocyte differentiation, we investigated the relationship between the binding of key transcription factors and m6A levels. We discovered that the association between m6A levels and transcription factor binding strength varied across different transcription factors. For instance, the binding strength of NANOG, a stem cell marker, showed no correlation with m6A levels at either D0 or D15 (Additional file 1: Fig. S6). In contrast, the binding strength of POU5F1, another stem cell marker, was related to m6A levels at D0 (Additional file 1: Fig. S6). Similarly, the binding strength of key cardiomyocyte factors correlated with m6A levels to some extent (Additional file 1: Fig. S6). This evidence further suggests that the relationship between accessibility and m6A may be a factor influencing cardiomyocyte differentiation.

To further examine the impact of chromatin accessibility on m6A during cardiomyocyte differentiation, we analyzed changes in both chromatin accessibility and m6A levels throughout the differentiation stages. We categorized genes into five groups based on their distinct patterns of change: (1) Genes with no changes in either chromatin accessibility or m6A levels. (2) Genes with only changes in chromatin accessibility while m6A levels remained constant. (3) Genes with only changes in m6A levels while chromatin accessibility remained constant. (4) Genes with contrasting trends of change in chromatin accessibility and m6A levels. (5) Genes with similar trends of change in both chromatin accessibility and m6A levels. (Fig. 5C). We sought to understand the primary roles of the different gene categories and performed GO enrichment analysis for each stage (Fig. 5D, Additional file 1: Fig. S7). We found that genes with changing m6A levels alone were related to cardiomyocyte differentiation throughout the entire process. In contrast, genes with only chromatin accessibility changes were mainly involved in basic cellular functions, such as cell metabolism. Interestingly, genes with concurrent chromatin accessibility and m6A level changes shared functions with those displaying m6A changes alone during the early differentiation stages. Both categories were associated with cell fate determination and promoting cell differentiation. However, in later stages, these genes resembled those with independent chromatin accessibility changes, primarily responsible for basic cellular functions (Additional file 1: Fig. S7). Comparing the correlation between chromatin accessibility and m6A level changes revealed a similar phenomenon. During early and middle stages of differentiation, genes with larger changes in accessibility tended to exhibit higher m6A levels, whereas during later stages, the opposite trend was observed (Fig. 5E). This suggests that m6A regulation during differentiation occurs through different mechanisms. In the early and middle stages, chromatin accessibility and m6A modification jointly regulate differentiation. However, in later stages, m6A no longer appears to regulate differentiation in concert with chromatin accessibility. Lastly, we examined whether the relationship between chromatin accessibility and m6A modification levels remained consistent throughout differentiation. By overlapping different gene classes at each stage, we found that only a small number of genes exhibited a consistent relationship between chromatin accessibility and m6A level changes across all stages (Fig. 5F, Additional file 1: Fig. S8). This indicates that genes are regulated by varying chromatin states and m6A modification levels during different differentiation stages, which aligns with our previous findings.

## Discussion

During cell differentiation, the transcriptome undergoes significant changes, primarily regulated by various epigenetic factors, such as DNA methylation and histone modification. Recent studies have demonstrated that m6A modification on mRNA is essential for regulating gene expression during cell differentiation [25, 35]. However, due to technical limitations in m6A detection, there have been no reports on the dynamic process of m6A during stem cell differentiation into cardiomyocytes [39]. This study primarily offers a reliable m6A profiling map using the latest m6A detection method, laying the groundwork for future research on the role of m6A during cardiomyocyte differentiation [31].

We identified potential target genes for METTL14, RBM15, and ALKBH5 in our study. However, it should be noted that gene expression and m6A levels can be influenced by various factors, and additional target genes may exist. The genes not discovered in our study could be involved in more complex regulatory networks, and further research on these genes could provide a more comprehensive understanding of the role of m6A in cardiomyocyte differentiation.

Our results indicate that the combined effects of m6A and chromatin accessibility are stage-specific, but the underlying mechanism remains unclear. Studies have shown a connection between m6A modification and certain histone modifications. Histone modification also plays a crucial role during cardiomyocyte differentiation. Thus, further research on the relationship between histones and m6A could potentially shed light on the association between m6A and chromatin accessibility. Additionally, it is essential to consider DNA methylation. There is evidence that m6A modification is linked to 5-methylcytosine (5mC) modification, and DNA modification also influences cardiomyocyte differentiation progress [40, 41]. Hence, investigating the relationship between m6A and chromatin accessibility in terms of DNA methylation might provide valuable insights into the intricate regulatory mechanisms. Further studies in these directions could significantly contribute to a deeper understanding of the interplay between m6A, chromatin accessibility, and other epigenetic factors during cardiomyocyte differentiation.

In summary, our comprehensive investigation into the role of m6A in cardiomyocyte differentiation has provided valuable insights into its regulatory mechanisms, particularly in relation to chromatin accessibility. Our findings contribute to a better understanding of cardiomyocyte differentiation and hold promise for addressing challenges related to cardiovascular diseases in the future.

## Methods

### Cell Culture

H1 human embryonic stem cells (hESCs) (obtained from the WiCell Research Institute) were grown on Matrigel (BD, 354277)-coated 6-well plates in E8 medium (Stem Cells Technology, 05940) at 37 ℃ with 5% CO_2_. Cells were passaged every 3–4 days using 0.5 mM EDTA (ThermoFisher, AM9260G) in Dulbecco’s phosphate buffered saline (DPBS) without Ca^2+^ or Mg^2+^ (Gibco, 14190136) at 37 ℃. 5 μM Rho kinase inhibitor Y-27632 (Selleck, S1049) was added for the first 24 h after passaging. The E8 medium was changed every day.

### Cardiomyocyte (CM) differentiation

Undifferentiated hESCs cultured in E8 medium were dissociated into single cell suspension by Accutase (Stem Cells Technology, 7920) and reseeded onto Matrigel-coated 24-well plate at a density of 105 cells/well in E8 medium containing 10 μM Y-27632. When cells reach ~ 80% confluence 2–3 days after plating, CM differentiation was initiated by switching to the differentiation medium named E8 basal + Lip (DMEM/F-12 (Gibco, 11330032) supplemented with 50 U ml-1 Penicillin–Streptomycin (Gibco, 15070063), Chemically Defined Lipid Concentrate (1:100, Gibco, 11905031), 10.7 μg ml-1 holo-Transferrin human (Sigma, T0665), 71 μg ml-1 L-Ascorbic acid (Sigma, A8960), 14 ng ml-1 Sodium selenite (Sigma, S5261)). 5 μM CHIR99021 (Selleck, S1263) or 3 μM IWP2 (Selleck, S7085) was added into the cardiac differentiation medium from days 0–1 and days 2–5, respectively. 3 μg ml-1 heparin was added into the cardiac differentiation medium from days 1–7. 20 μg ml-1 Insulin (Sigma, 91077C) was added into the cardiac differentiation medium from day 7 onward and renewed every 2–3 days.

### Sample collection

Cell samples were captured at time points corresponding to stage-specific transitions in cell state including pluripotency stem cell (differentiation Day 0), mesoderm (Day 2), cardiac progenitor cells (Day 5), and cardiomyocytes (Day 15). Cells were dissociated into single cell using Accutase for 5 min at 37 °C, washed twice with ice-cold wash buffer (DPBS containing 2% FBS). Total RNA was extracted using the NucleoZol reagent (ThermoFisher, 15596026) and quantified by a NanoDrop spectrophotometer (ThermoFisher). Then mRNA was purified from total RNA using Dynabeads™ mRNA Purification Kit (ThermoFisher, 61,006).

### MeRIP-seq

The MeRIP-seq protocol builds based on some previous protocols. Briefly, RNA samples were first fragmented to 150 nt by Fragmentation Reagent (ThermoFisher, #AM8047), and then the purified fragmented RNA was end-repaired using T4 Polynucleotide Kinase (ThermoFisher, #EK0031). Next, each sample was ligated with different 3′ adapters with barcodes that had been adenylated in advance using 5' DNA Adenylation Kit (NEB, #E2610) with T4 RNA ligase 2, truncated KQ (NEB, #M0373). After ligation, Lambda Exonuclease (NEB, #M0262) was used to remove excess adapters. After the purified products were mixed, 1/10 was taken out as input and temporarily stored at − 20 °C, and the remaining products were subjected to IP in the next step. ProteinG beads (ThermoFisher, #10004D) were washed twice in reaction buffer (150 mM NaCl, 10 mM Tris–HCl (PH7.4), 0.01% Igepal CA-630) and incubated with m6A antibody (CST, #56,593) at 4 ℃ on rotator for 40–60 min. The pretreated beads were washed twice in reaction buffer and then incubated with samples at 4 ℃ on rotator for 2 h. After the IP incubation, samples were washed with reaction buffer, low-salt buffer (50 mM NaCl, 10 mM Tris–HCl (PH7.4), 0.01% Igepal CA-630) and high-salt buffer (500 mM NaCl, 10 mM Tris–HCl (PH7.4), 0.01% Igepal CA-630). Each wash step was conducted 3 times at 4 ℃ on rotator. RNA was eluted from beads with buffer RLT (QIAGEN, #79,216) and a small amount of sample could be taken for qPCR. Then 5′ adapters were ligated to samples using T4 RNA Ligase 1 (NEB, #M0204). After ligation, the product was reverse-transcribed using HiScript III 1st Strand cDNA Synthesis Kit (Vazyme, #R312) to generate a cDNA, and the library amplification was performed using 2 × KAPA HiFi Hot Start Ready Mix (KAPA Biosystems, #KK2602 7958935001).

### RNA-seq data analysis

Following quality controls (performed with FastQC v0.11.5), reads were aligned to the hg19 genome using hisat2 v2.1.0 [42]. Gene expression levels were quantified by Featurecounts v1.6.2 [43] and DESeq2 v1.24.0 [44]. Genes with low expression or undetectable in all four samples were filtered out, and 21,269 genes were retained for downstream analysis. The discrete score of a gene in Fig. 3A is calculated as the standard deviation of its expression across four different stages of differentiation.

### Quantitative MeRIP-seq data analysis

Paired-end reads of MeRIP-seq were demultiplexed into individual samples with fastq-multx v1.4.3 [45]. Demultiplexed reads were mapped to the hg19 genome using hisat2 v2.1.0. with default parameters. Reads with duplicate UMI were removed using UMI_tools v1.0.1 [46]. Peak calling was performed by MACS2 with the parameters “—nomodel”. Peaks with input reads less than 5 in any sample were filtered out and 10,020 peaks were retained. The reads coverage was normalized to the total reads in each sample to ensure comparability across samples. The differential m6A peaks were defined as those showing a fold change in coverage > 1.2 between two samples and a p-value < 0.05 using the chi-square test. Peaks were annotated to genes using ChIPpeakAnno_3.24.2 [47]. m6A level of a gene was defined as the ratio of the IP reads to input reads in the peaks on gene. Motif enrichment was performed by HOMER [48]. Metagene coverage density was calculated by aggregating the normalized coverage of each gene.

### ATAC-seq data analysis

Reads were mapped with bwa v0.7.17 to hg 19 genome using default parameters. Mapped reads which MAPQ > 30 were kept. Picard-tools v1.129 was use to remove duplicate reads. Peak calling was performed by MACS2 with the parameters “—nomodel -q 0.01 —shift 100 —extsize 200”. Peaks were annotated to genes using ChIPpeakAnno_3.24.2. Quantification was performed by Featurecounts v1.6.2 and DESeq2 v1.24.0.

### ChIP-seq data analysis

Reads were mapped with bowtie v1.2.1.1 [49] (for reads length <  = 50) or bowtie2 v2.3.4 [50] (for reads length > 50) to hg 19 genome using default parameters. Reads with MAPQ < 30 were filtered out. Samtools markdup [51] was use to remove duplicate reads. Peaks were called with MACS2 using the parameters “—nomodel -q 0.05 —extsize 150”. Peaks were annotated to genes using ChIPpeakAnno_3.24.2. Quantification was performed by Featurecounts v1.6.2 and DESeq2 v1.24.0.

### Correlation analysis

The correlation between expression and m6A level and the correlation between accessibility and m6A level were measured by Pearson correlation coefficient. Genes with correlation coefficients in the top 5% and bottom 5% were classified as positively and negatively correlated genes (pos cor genes and neg cor genes), respectively. Genes with correlation coefficients between −0.1 and 0.1 for expression/accessibility and m6A level were classified as not correlated genes (not cor genes).

### GO enrichment analysis

The visualization of GO enrichment was performed by Metascape [52] and R. P-value < 0.05 was considered as statistically significant.

### Supplementary Information

**Additional file1: Figure S1.** Expression level changes during cardiomyocyte differentiation. **A** Heatmap of expression levels during cardiomyocytes differentiation. **B** Heatmap of expression levels of some key factors during cardiomyocytes differentiation. **Figure S2.** GO enrichment analysis results of genes under different clusters in Figure 2A. **Figure S3.** Correlation between gene m6A levels and expression levels. **A** Dot plot show a weak negative correlation between gene m6A levels and expression levels during differentiation. **B** Comparison of gene expression levels among different m6A levels. Genes were categorized into three groups based on their m6A levels: low (m6A level < 1.5), medium (1.5 < m6A level < 4), and high (m6A level > 4). ** represents p-value<0.01. **Figure S4.** Correlation analysis between the expression of potential target genes associated with cardiomyocyte differentiation (regulated by METTL14, RBM15, and ALKBH5) and the expression levels of m6A readers. The correlation was assessed using the Pearson correlation coefficient. **Figure S5.** **A** The correlation between reader expression and m6A does not affect the accessibility of the corresponding gene. **B** The reader responds differently to genes with different degrees of accessibility. neg/not/pos cor: genes whose m6A levels or accessibility are negatively/not/positively correlated with the expression of m6A-related readers. **Figure S6.** Comparison of binding levels of key transcription factors with different m6A level in D0 and D15. **A** Binding levels of key transcription factors in stem cells. **B** Binding levels of key transcription factors in cardiomyocytes. Genes were categorized into three groups based on their m6A levels: low (m6A level < 1.5), medium (1.5 < m6A level < 4), and high (m6A level > 4). * represents p-value<0.05, Wilcoxon test. **Figure S7.** GO enrichment analysis results of genes under four groups base on their m6A level and accessibility changes: **A** genes with discordant changes, **B** genes with concordant changes, **C** genes with only m6A changes, and **D** genes with only accessibility changes

## Data Availability

All data generated for this paper have been deposited at NCBI’s Gene Expression Omnibus (GEO) under accession number GSE231549. The ATAC-seq datasets analyzed for this paper are available under GEO accession number GSE106689 [38]. The ChIP-seq datasets analyzed for this paper are available under GEO accession number GSE61475 [53], GSE85631 [54] and GSE89457 [55].

## Abbreviations

m6A: N6-methyladenosine
GO: Gene ontology
