# Supplementary material for: Co-effects of m6A and chromatin accessibility dynamics in the regulation of cardiomyocyte differentiation
Source: Epigenetics Chromatin. 2023 Aug 11;16:32. doi: 10.1186/s13072-023-00506-6 (PMC10416456; doi:10.1186/s13072-023-00506-6)
Supplement: Supplementary file 1 — Additional file1: Figure S1. Expression level changes during cardiomyocyte differentiation. A Heatmap of expression levels during cardiomyocytes differentiation. B Heatmap of expression levels of some key factors during cardiomyocytes differentiation. Figure S2. GO enrichment analysis results of genes under different clusters in Figure 2A. Figure S3. Correlation between gene m6A levels and expression levels. A Dot plot show a weak negative correlation between gene m6A levels and expression levels during differentiation. B Comparison of gene expression levels among different m6A levels. Genes were categorized into three groups based on their m6A levels: low (m6A level < 1.5), medium (1.5 < m6A level < 4), and high (m6A level > 4). ** represents p-value<0.01. Figure S4. Correlation analysis between the expression of potential target genes associated with cardiomyocyte differentiation (regulated by METTL14, RBM15, and ALKBH5) and the expression levels of m6A readers. The correlation was assessed using the Pearson correlation coefficient. Figure S5. A The correlation between reader expression and m6A does not affect the accessibility of the corresponding gene. B The reader responds differently to genes with different degrees of accessibility. neg/not/pos cor: genes whose m6A levels or accessibility are negatively/not/positively correlated with the expression of m6A-related readers. Figure S6. Comparison of binding levels of key transcription factors with different m6A level in D0 and D15. A Binding levels of key transcription factors in stem cells. B Binding levels of key transcription factors in cardiomyocytes. Genes were categorized into three groups based on their m6A levels: low (m6A level < 1.5), medium (1.5 < m6A level < 4), and high (m6A level > 4). * represents p-value<0.05, Wilcoxon test. Figure S7. GO enrichment analysis results of genes under four groups base on their m6A level and accessibility changes: A genes with discordant changes, B genes with concordan [file 13072_2023_506_MOESM1_ESM.docx]

**Additional file 1**


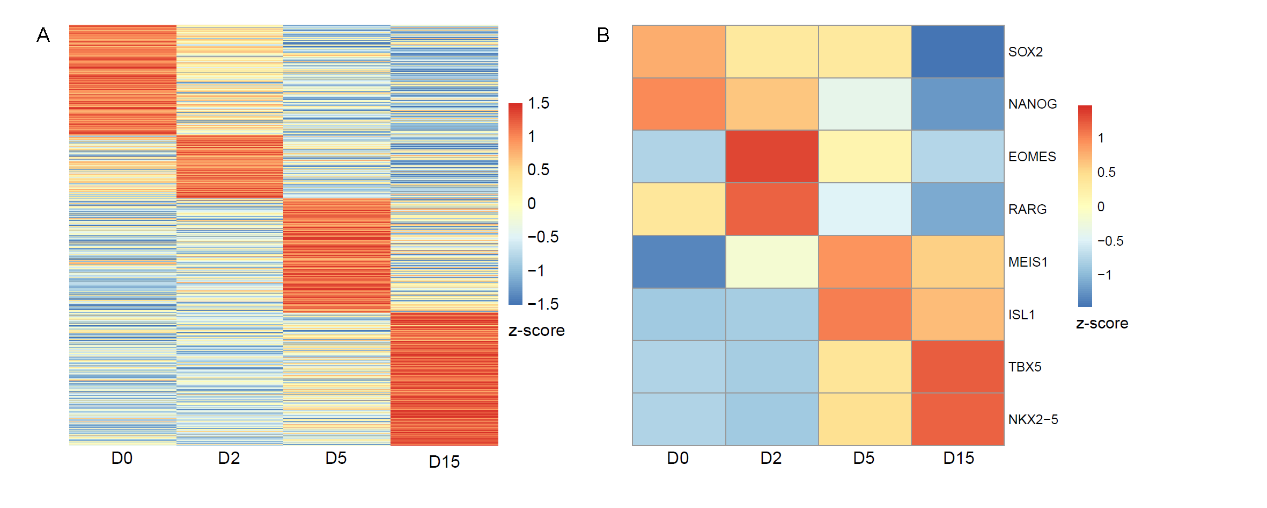


**Additional file 1** Figure 1 Expression level changes during cardiomyocyte differentiation. (A) Heatmap of expression levels during cardiomyocytes differentiation. (B) Heatmap of expression levels of some key factors during cardiomyocytes differentiation.


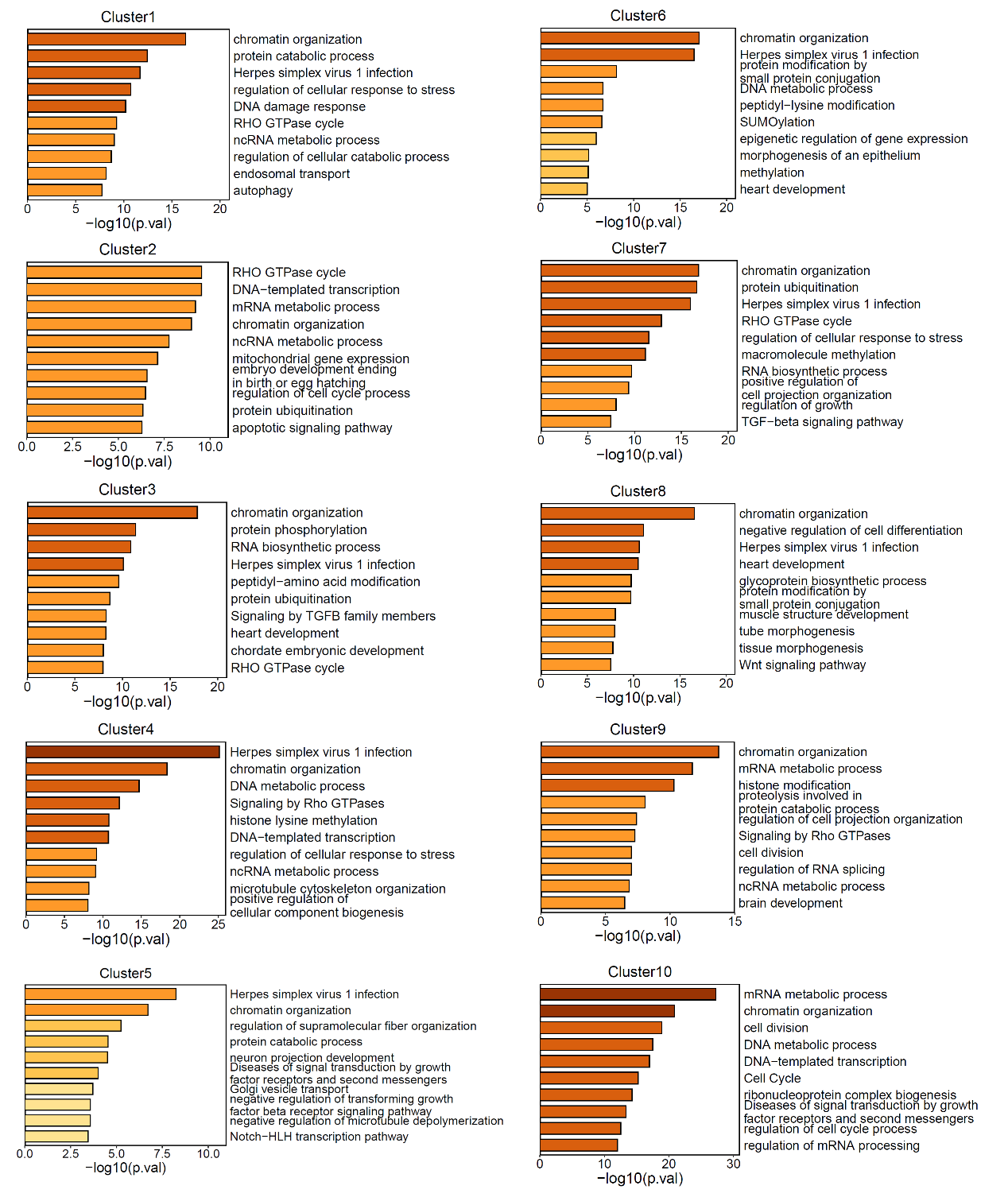


**Additional file 1** Figure 2 GO enrichment analysis results of genes under different clusters in Figure 2A.


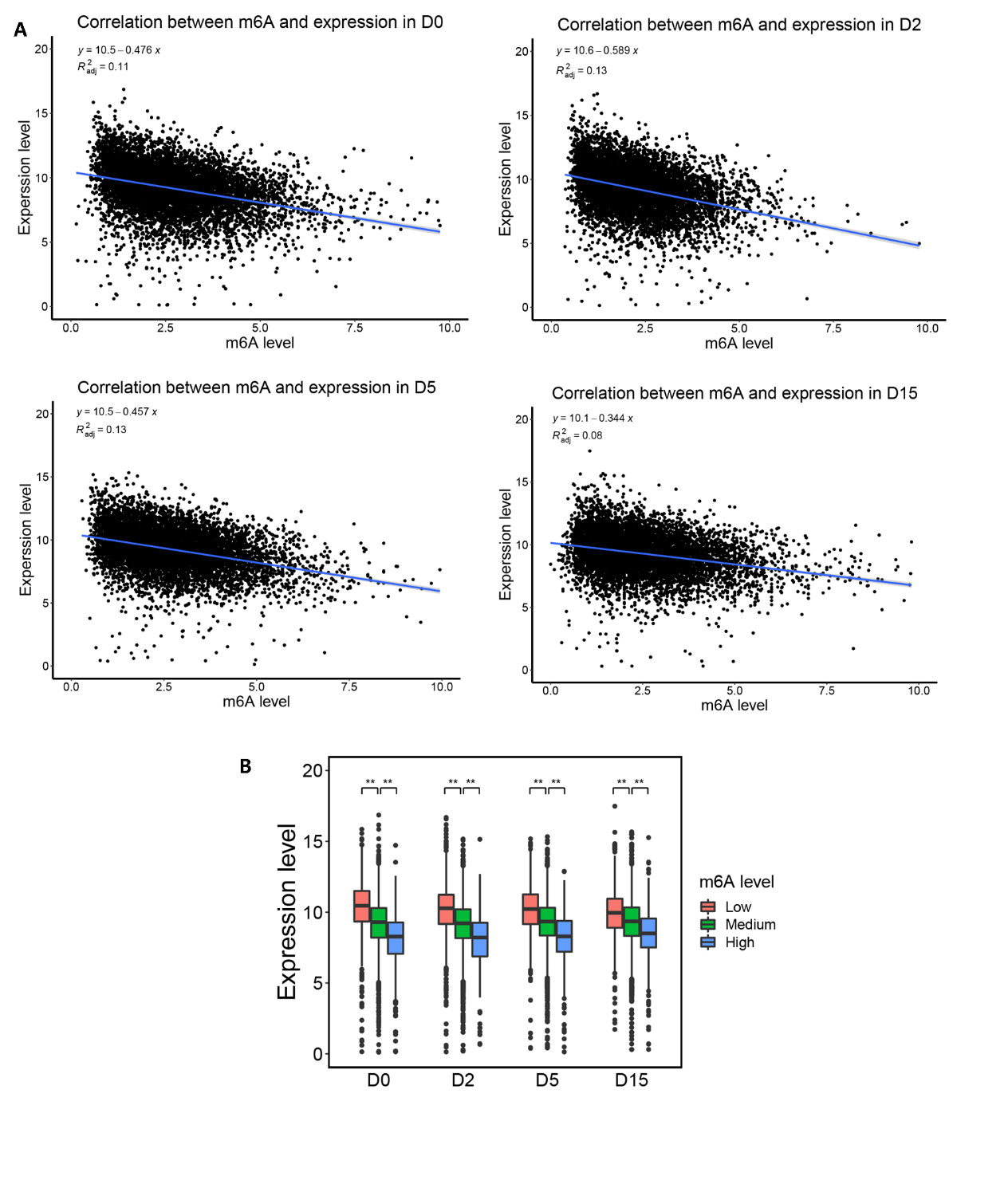


**Additional file 1** Figure 3 Correlation between gene m6A levels and expression levels. (A) Dot plot show a weak negative correlation between gene m6A levels and expression levels during differentiation. (B) Comparison of gene expression levels among different m6A levels. Genes were categorized into three groups based on their m6A levels: low (m6A level < 1.5), medium (1.5 < m6A level < 4), and high (m6A level > 4). ** represents p-value<0.01.


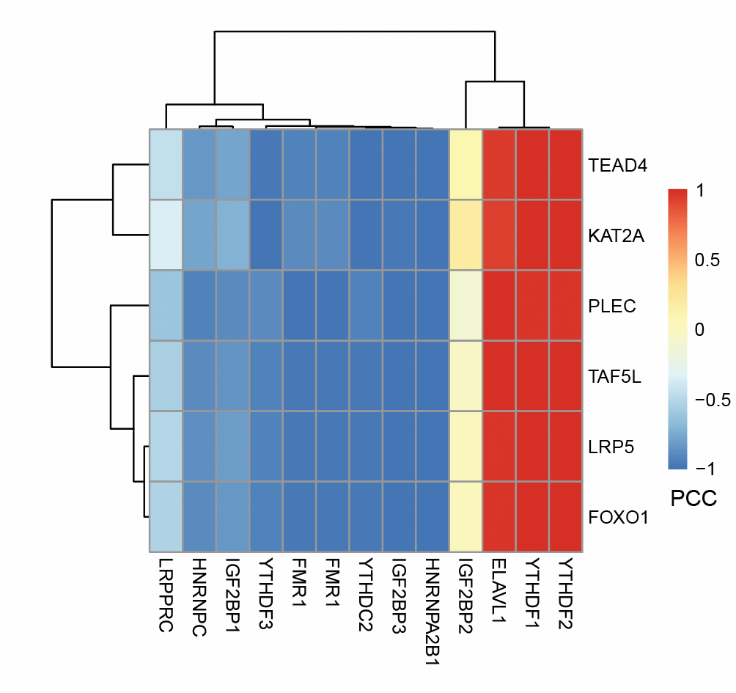


**Additional file 1** Figure 4 Correlation analysis between the expression of potential target genes associated with cardiomyocyte differentiation (regulated by METTL14, RBM15, and ALKBH5) and the expression levels of m6A readers. The correlation was assessed using the Pearson correlation coefficient.


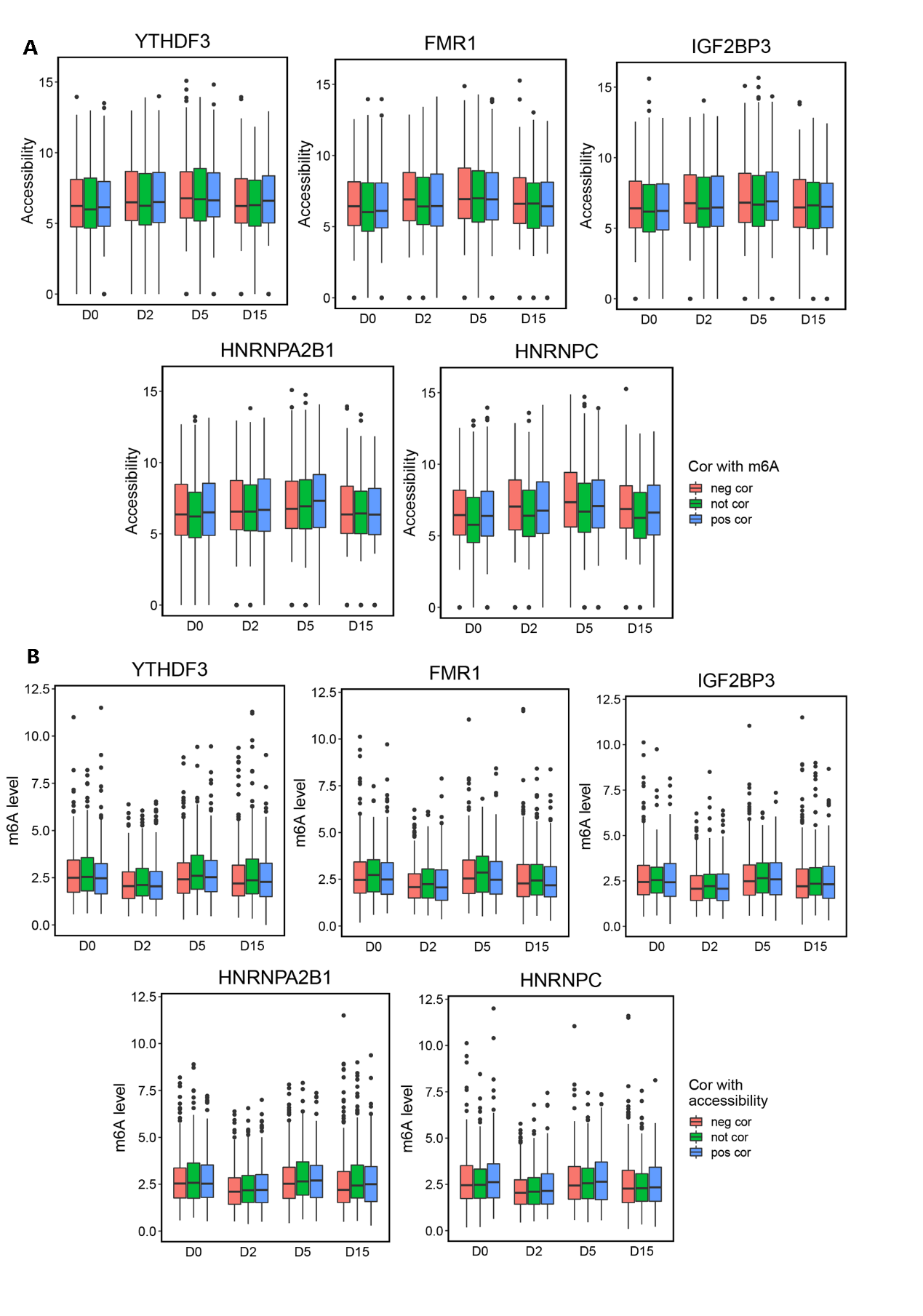


**Additional file 1** Figure 5 (A) The correlation between reader expression and m6A does not affect the accessibility of the corresponding gene. (B) The reader responds differently to genes with different degrees of accessibility. neg/not/pos cor: genes whose m6A levels or accessibility are negatively/not/positively correlated with the expression of m6A-related readers.


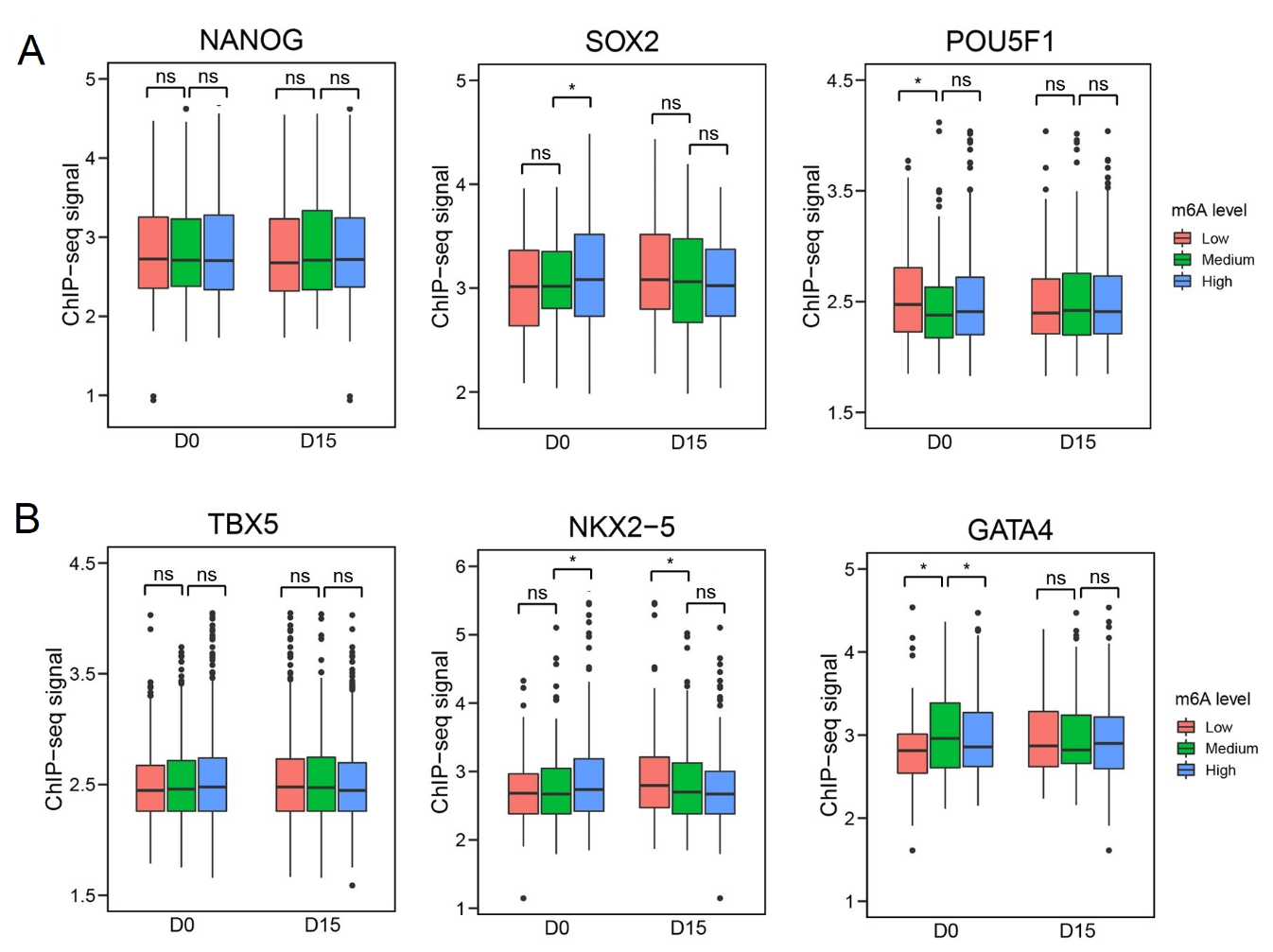


**Additional file 1** Figure 6 Comparison of binding levels of key transcription factors with different m6A level in D0 and D15. (A) Binding levels of key transcription factors in stem cells. (B) Binding levels of key transcription factors in cardiomyocytes. Genes were categorized into three groups based on their m6A levels: low (m6A level < 1.5), medium (1.5 < m6A level < 4), and high (m6A level > 4). * represents p-value<0.05, Wilcoxon test.


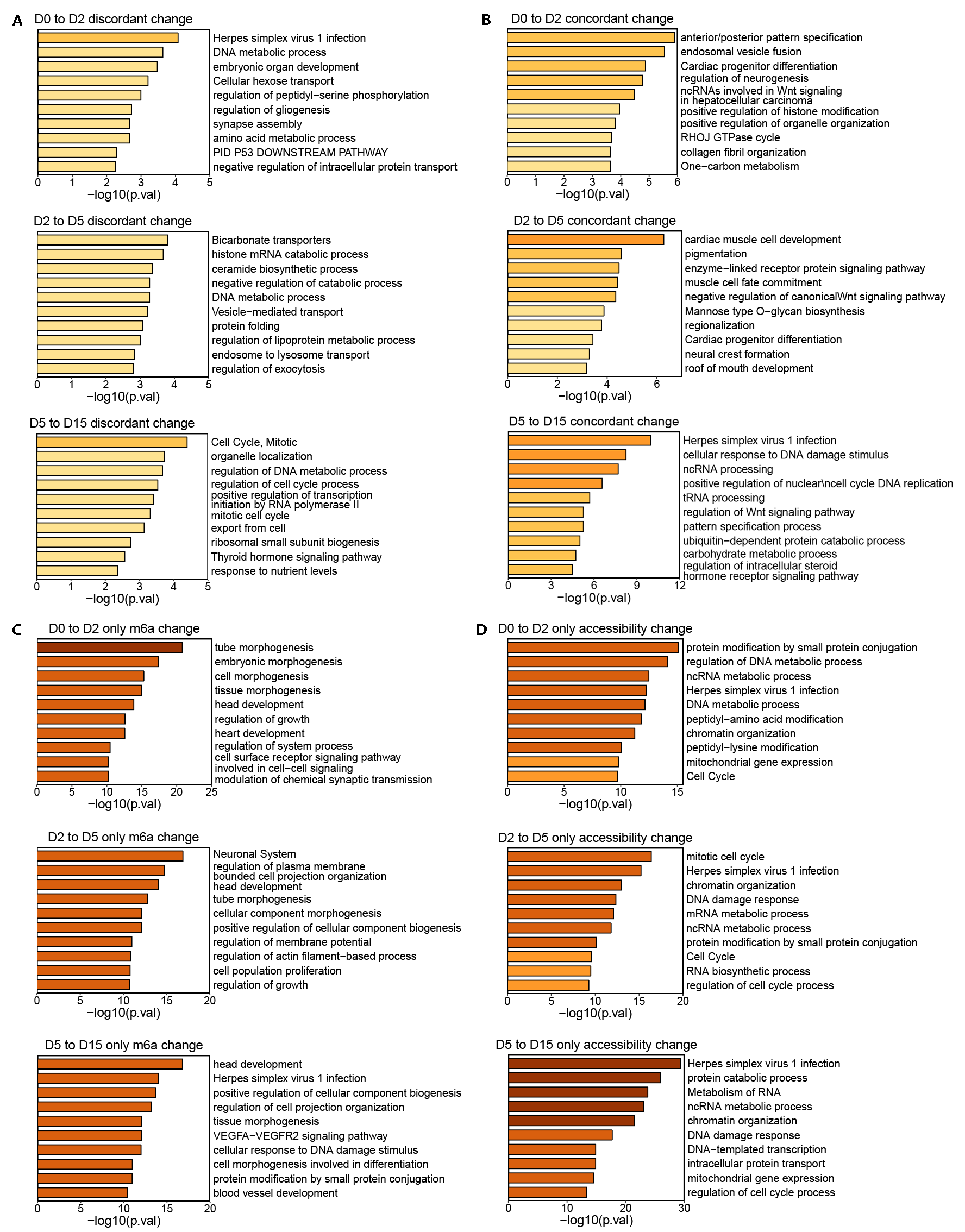


**Additional file 1:** Figure 7 GO enrichment analysis results of genes under four groups base on their m6A level and accessibility changes: (A) genes with discordant changes, (B) genes with concordant changes, (C) genes with only m6A changes, and (D) genes with only accessibility changes.


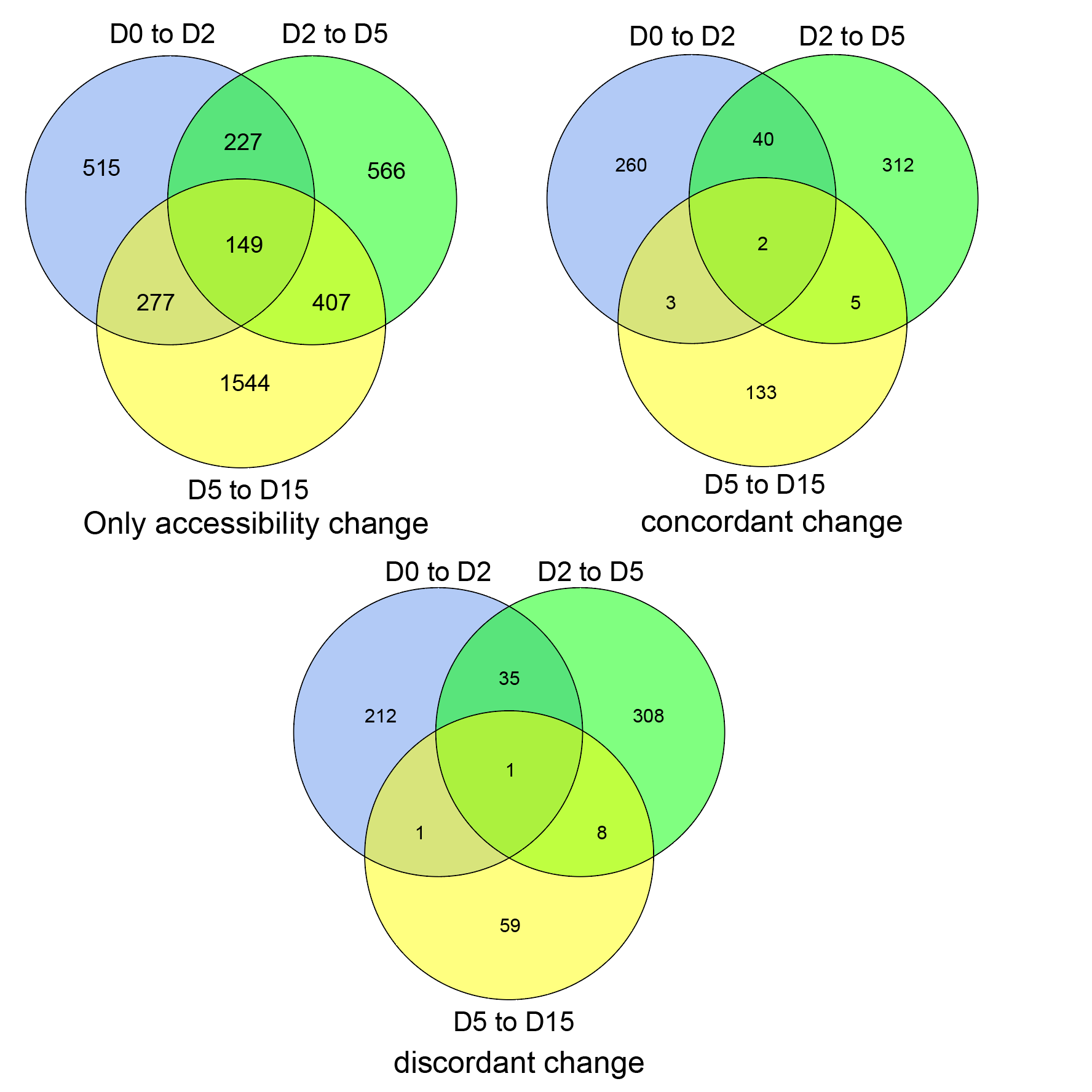


Supplementary Figure 8 Overlap of genes in different categories defined in Figure 5C across different differentiation stages.
